# General Anesthesia Does Not Have Persistent Effects on Attention in Rodents

**DOI:** 10.3389/fnbeh.2019.00076

**Published:** 2019-04-17

**Authors:** Viviane S. Hambrecht-Wiedbusch, Katherine A. LaTendresse, Michael S. Avidan, Amanda G. Nelson, Margaret Phyle, Romi E. Ajluni, George A. Mashour

**Affiliations:** ^1^Department of Anesthesiology, University of Michigan, Ann Arbor, MI, United States; ^2^Center for Consciousness Science, University of Michigan, Ann Arbor, MI, United States; ^3^Department of Anesthesiology, Washington University, St. Louis, MO, United States

**Keywords:** 5-Choice Serial Reaction Time Task, accuracy, cognitive dysfunction, delirium, isoflurane, ketamine, omission, premature response

## Abstract

**Background:** Studies in animals have shown that general anesthesia can cause persistent spatial memory impairment, but the influence of anesthetics on other cognitive functions is unclear. This study tested whether exposure to general anesthesia without surgery caused a persistent deficit in attention in rodents.

**Methods:** To evaluate whether anesthesia has persistent effects on attention, rats were randomized to three groups. Group A was exposed for 2 h to isoflurane anesthesia, and tested the following seven days for attentional deficits. Group B was used as a control and received room air before attentional testing. Since there is some evidence that a subanesthetic dose of ketamine can improve cognition and reduce disorders of attention after surgery, rats in group C were exposed to isoflurane anesthesia in combination with a ketamine injection before cognitive assessment. Attention was measured in rats using the 5-Choice Serial Reaction Time Task, for which animals were trained to respond with a nose poke on a touchscreen to a brief, unpredictable visual stimulus in one of five possible grid locations to receive a food reward. Attention was analyzed as % accuracy, % omission, and premature responses.

**Results:** Evaluating acute attention by comparing baseline values with data from the day after intervention did not reveal any differences in attentional measurements. No significant differences were seen in % accuracy, % omission, and premature responses for the three groups tested for 7 consecutive days.

**Conclusion:** These data in healthy rodents suggest that general anesthesia without surgery has no persistent effect on attention and the addition of ketamine does not alter the outcome.

## Introduction

Every year more than 300 million patients undergo surgery worldwide, the majority with general anesthesia (Weiser et al., 2015). Studies in humans and animals suggest the possibility of immediate and/or persistent cognitive impairments attributable to general anesthesia. In rodents, for example, general anesthesia alone can cause disorders in spatial cognition (Culley et al., 2004a,b) as well as impaired learning and memory (Culley et al., 2003; Jevtovic-Todorovic et al., 2003; Bianchi et al., 2008; Lin and Zuo, 2011). These preclinical data raise the question of whether general anesthesia in humans is responsible for post-operative cognitive dysfunctions such as delirium. Delirium is a complex syndrome characterized by acute and fluctuating cognitive impairment that prominently involves attention (Gupta et al., 2008; American Psychiatric Association, 2013; World Health Organization, 2018). Delirium is associated with short- and long-term consequences including increased risk of falls, diminished quality of life, prolonged hospital stay, higher costs for patients and hospitals, and increased mortality rates (Ely et al., 2001a,b; McCusker et al., 2002; Inouye, 2006; Siddiqi et al., 2006; Leslie et al., 2008; Witlox et al., 2010; Rudolph and Marcantonio, 2011). However, most basic science studies of general anesthesia and cognition in animals have focused on spatial learning and memory, which has questionable relevance to the human phenotype of delirium. Thus, in order to assess whether it is biologically plausible that exposure to general anesthesia alone could cause delirium in the days following exposure, we tested the effects of isoflurane on attention (measured with the 5-Choice Serial Reaction Time Task; Robbins, 2002) for a 1 week period.

## Materials and Methods

### Animals

This study was carried out in accordance with the recommendations of the Guide for the Care and Use of Laboratory Animals (National Academies Press, 8th Edition, Washington, DC, 2011) and the Institutional Animal Care and Use Committee (IACUC) supported by the Animal Care and Use Office. The protocol was approved by the IACUC. Male, 2–3 months old Sprague-Dawley rats (*n* = 18) were purchased from Envigo (Michigan, United States), housed in pairs in identical chambers under a 12:12 h light:dark cycle, and were allowed a 7 day acclimation period before the study.

### Attention Assessment With the 5-Choice Serial Reaction Time Task

**FIGURE 1:**
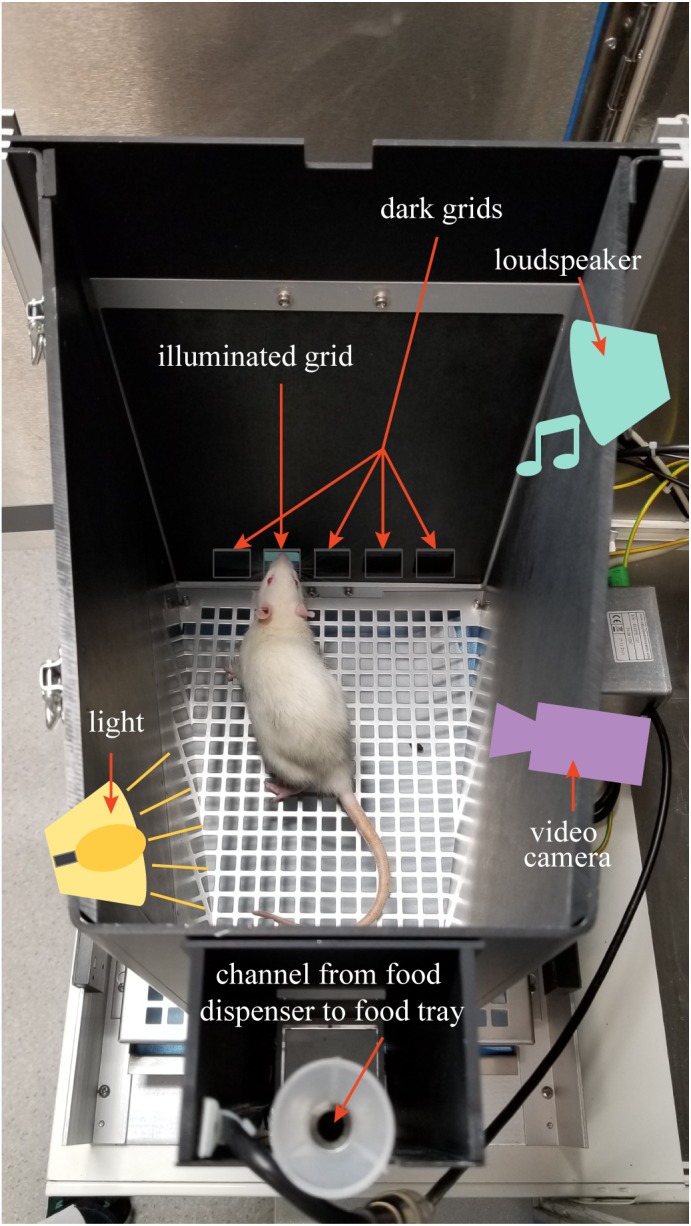
The figure shows the 5-CSRTT chamber setup. Loudspeaker, video camera, light source, and food dispenser are above a lid covering the testing chamber.

The Bussey-Saksida Touchscreen System from Lafayette Instruments (Lafayette, IN, United States) was used for the 5-Choice Serial Reaction Time Task (5-CSRTT) (Bussey et al., 2008). This task measures attention and requires the rodent to respond to a brief visual stimulus presented randomly in one of five locations. This task has high translational value since it is adapted from the human continuous performance task (Robbins, 2002). First, all animals were food restricted to 80–90% of their free feed body weight to motivate them to perform in the 5-CSRTT, but had ad lib access to water. Animals were weighed daily to ensure overall health. Sucrose pellets (45 mg, unflavored, product number F06233, Bio-Serv, Flemington, NJ, United States) provided a reward for performing the task. The 607.55 cm^2^ (94.17 square inch) chamber was set with a touchscreen on one side covered with a black plastic board keeping five grids open for possible light stimuli. The food tray was situated on the opposite side of the chamber. An infra-red video camera was installed above the chamber, which allowed video recording of task performance (Figure 1). The task was performed in the dark to give a better contrast for light stimulus.

### Conditioning and Testing Schedule for the 5-CSRTT

The animals were trained according to the CAM 5-CSRTT protocol (CAM Rat Touch 5CSRT ABETT II Manual V2_2.pdf, Lafayette Instruments Co., IN, United States). This protocol is run in darkness to allow better contrast for the illuminated grid. In short, rats were habituated to the chamber and were allowed to discover the food tray, which was filled with several sucrose pellets (Habituation, 30 min) and which was on the opposite side of the chamber as the touchscreen. Animals were trained the following days to use their nose to touch one of the five possible illuminated screen grids to receive a food reward. Only one grid was illuminated at a time (the rest stayed dark) and the position of the illuminated grid was chosen pseudorandomly, meaning that the stimulus was not displayed in the same position more than three times in a row. After a delay (30 s), the grid turned dark and one sucrose pellet was delivered into the then illuminated food tray. The nose poke into the tray turns off the light and starts the inter-trial interval (ITI, 5 s). After the ITI, another grid is illuminated. If an animal touches the correct (illuminated) grid while it is displayed, the grid became dark and a tone (1000 ms) was played while, simultaneously, three sucrose pellets are delivered into the food tray. Reward collection initiated the ITI (Initial Touch, 100 trials over max. 60 min). During the next phase, the rat must learn to touch the correct grid to elicit a tone (1000 ms) combined with the release of a sucrose pellet into the then illuminated food tray, indicating a correct answer. There is no response if the animal touches a dark grid. Entry into the food tray turns the light off and starts the ITI (Must Touch, 100 trials over max. 60 min). For the following conditioning level, the rat needs to learn to initiate a trial. Therefore, the animal received one food pellet into the illuminated tray at the beginning of the session. The rat must then poke its nose into the food tray before a stimulus is presented on the touchscreen. As before, the animal needs to touch the correct grid to receive a reward (as described under the Must Touch session above). Retrieving the reward turns the tray light off and starts the ITI. After the 5 s ITI period the light in the food tray turned on and the animal needed to initiate the next trial by poking its nose into and out of the food tray before the next image was displayed (Must Initiate, 100 trials over max. 60 min). In the next phase, the animals were trained as described above but when the animal touched an incorrect (not illuminated) grid, the light on top of the chamber was automatically turned on indicating a time out time (5 s) during which the animal needed to wait and did not receive a reward (Punish Incorrect, 100 trials over max. 60 min). The same time out with no reward occurred during the later stages if an animal did not react during the limited hold time after a grid light illumination (the time the animal had to respond to the correct grid) or when an animal reacted prematurely to the stimulus (during the 5 s delay between initiating the next trial and the illumination of one grid). After a time out, the animal needed to initiate the next trial. A trial ended when either a reward was collected, an incorrect response was made, or the time out began following an omission or premature response. Time spent in the chamber depended on the training session and how well the animal performed. Sessions were deemed complete when the animal either fulfilled the trial runs or when the time set for the trial was over, whichever occurred first. From Initial Touch to Punish Incorrect, animals moved to the next training level when the number of correct responses was 80 or higher. Every animal received only one training session per day. After the animals had, for at least 2 days, 80 or more correct responses at the Punish Incorrect training level, rats were conditioned to reach the baseline level for the testing phase. This was done in eight stages, during which everything was done the same way as during the Punish Incorrect training phase. However, the limited hold time (60, 30, 20, 10, 5, 5, 5, 5 s) and the stimulus duration (60, 30, 20, 10, 5, 2.5, 1.5, 1 s) were reduced with every stage, ensuring that the animal needed to pay more attention. In addition, omissions and premature responses ended in a time out phase. To move to the next stage, an animal needed to meet the learning criteria of accuracy >80% and omission <20%. After the animal successfully learned the eighth stage, it was randomly assigned to one of three treatment groups (see below in section Experimental Groups). A baseline session was performed 1 day after reaching the eighth stage followed by treatment the day after that. The next day the animal performed the same stage (eighth) as for the baseline testing. From the following day on, the animal’s attention was tested using stages nine to fourteen, during which the stimulus duration was further reduced (0.9, 0.8, 0.7, 0.6, 0.5, 0.25 s), while limited hold time stayed at 5 s.

### Experimental Groups

**FIGURE 2:**
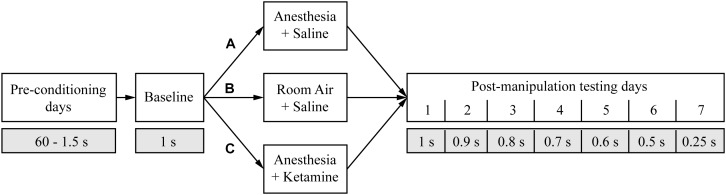
Study design. The timeline shows the different manipulations (white boxes) and the duration of the random light stimulus (gray boxes) for the entire experiment. The three different treatment groups are marked with A, B, and C.

After successfully learning the eighth conditioning stage (accuracy >80% and omission <20%), animals were randomly assigned to three groups (Figure 2). To evaluate the effect of anesthesia on attention, rats in group A (*n* = 6) were treated for 2 h with isoflurane anesthesia and underwent attentional testing the following 7 days. Animals in group B (*n* = 6) were used as controls and instead received room air for 2 h before undergoing the attentional tests for several days. There is some evidence that the anesthetic drug ketamine can improve cognition in the elderly (Hudetz et al., 2009a,c) possibly due to anti-inflammatory (Loix et al., 2011; Dale et al., 2012) and analgesic (Ahern et al., 2015) properties. Furthermore, administration of ketamine during isoflurane anesthesia accelerated recovery (Hambrecht-Wiedbusch et al., 2017), raising the question of whether ketamine as an adjunct could enhance cognition. Therefore, animals in group C (*n* = 6) were administered ketamine during isoflurane anesthesia followed by tests of attention. Rats were kept for 2 h under isoflurane anesthesia while also receiving an intraperitoneal injection (i.p.) of a subanesthetic dose of ketamine after about one-fourth of the total anesthesia time had elapsed. Rats were tested on attentional tasks starting the next day for 1 week. As a control for the stimulus of the injection itself in group C, animals in group A and B received a saline injection at the same time point.

### Experimental Design and Timeline

The day after the baseline measurement, an animal was placed in a modified Raturn (Bioanalytical Systems, Inc., IN, United States; for detailed modified Raturn design; see Hambrecht-Wiedbusch et al., 2017). The modification allowed the Raturn to be sealed such that inhaled anesthetics can be administered while the animal behaves freely. For animals in group A, the Raturn was sealed after about 30 min of acclimation time and filled with isoflurane in high-flow oxygen (10 L/min) until inlet and outlet monitors were consistently sensing 2.5% isoflurane for 2 min. After induction of general anesthesia, as defined by the loss of righting reflex, the animal was placed on its back and a temperature probe was inserted rectally through a door in the Raturn. Breathing rate and temperature were assessed every 12.5 min throughout the experiment. Isoflurane levels were maintained at 1.5% throughout the 2 h of anesthesia exposure (note that 1.4% is the minimum alveolar concentration for isoflurane in rodents; Pal et al., 2012). After 37.5 min under isoflurane anesthesia, the animal received an i.p. injection of 25 mg/kg saline. Isoflurane anesthesia was discontinued 82.5 min later and the animal was allowed time to recover (defined as the return of righting response). Afterward, the rat was returned to its home cage and fed. In group B, the animals followed the same timeline as rats in group A but were, as a control, only exposed to room air for 2 h (open Raturn); they received a saline injection at the same time point during the experiment as animals in groups A and C. Rats in group C followed the same protocol as animals in group A but were exposed to 2 h of isoflurane anesthesia with an i.p. ketamine (25 mg/kg) instead of a saline injection after 37.5 min of anesthesia time.

### Assessment of Attention

Since cognitive impairments like delirium in patients is characterized as an acute and fluctuating impairment of cognition and is not always apparent immediately after recovery from anesthesia because of the residual drug effects, animals were cognitively assessed for the 7 days starting after the day of experimentation. A decreasing stimulus duration (starting with 1 s on day one and ending with 0.25 s on day seven post-manipulation) was used to challenge the animal in order to counteract a possible ceiling effect if all days were tested with the same 1 s stimulus duration.

Attention was assessed by the two components of % accuracy and % omission, showing the ability of the animal to perform the task. In addition, we evaluated premature responses as a marker of concentration and restraint. Data were acquired automatically through the Abet II “CAM 5-Choice Serial Reaction Time Task” program provided by the Lafayette Instrument Company. All animals fulfilled the daily 60 trials.

**Percent accuracy** reflects how well the animal performed each trial and was calculated as follows:

$$\%\text{ accuracy}=\frac{\text{number of correct responses}}{{\text{total number of trials}}^{†}}\times 100$$

†: including number of omissions and premature responses

**Percent omission** is used to indicate how often the animal received a time out trial and therefore did not receive a reward. It was calculated as follows:

$$\%\;omission=\frac{\begin{array}{l} \text{number of trials not responded to within}\\ \text{the limited hold period} \end{array}}{{\text{total number of trials}}^{†}}\times 100$$

†: including number of omissions and premature responses

**Premature response** is a reaction at the start of a new trial and is regarded as a marker of impulsivity. It indicates the animal’s inability to concentrate and wait for the illumination of the grid light. Premature responses were also included in the total number of trials.

### Statistical Analysis

Results are presented as mean ± SD. All data were evaluated with input from the Center for Statistical Consultation and Research at the University of Michigan (Ann Arbor, MI, United States). To compare the acute effect of anesthesia within the different treatment groups, unpaired *t*-test with Welch correction was used to compare baseline values with day 1 post-manipulation data. Comparisons of baseline values for % accuracy, % omission, and premature responses were analyzed using one-way analysis of variance (ANOVA) followed by multiple comparison Tukey procedure for each treatment group. Percent accuracy, % omission, and premature responses were analyzed using a two-way, repeated measures ANOVA. All data were evaluated using GraphPad (Prism) version 7. A *p*-value less than 0.05 was considered statistically significant.

A direct comparison between baseline day and first day after anesthesia was used for each treatment group to evaluate the acute effect of anesthesia on attention, because on both of these days the animal received the same stimulus duration of 1 s. Since, starting with day two, the stimulus duration was continuously reduced, a direct comparison with baseline values was no longer appropriate.

## Results

### Acute Effect of Anesthesia on Attention

**FIGURE 3:**
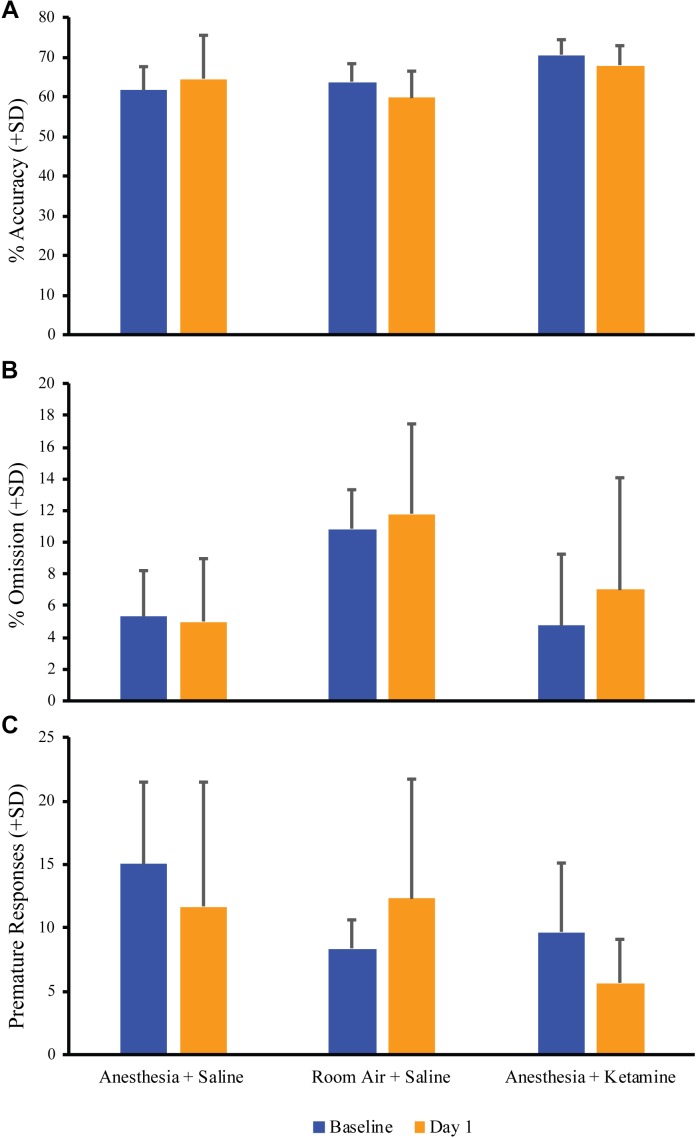
Acute effect of anesthesia on attention. Comparison of baseline and the day after manipulation (day 1) showed no significant difference for all treatment groups for % accuracy **(A)**, % omission **(B)**, and premature responses **(C)**.

Figure 3 depicts the comparison of baseline vs. day 1 post-anesthetic measurements for % accuracy, % omission, and premature responses for all treatment groups. Unpaired t-tests showed no significant difference between baseline and day 1 values for % accuracy (anesthesia+saline *p* = 0.5974, room air+saline *p* = 0.2700, anesthesia+ketamine *p* = 0.3303; Figure 3A), % omission (anesthesia+saline *p* = 0.7213, room air+saline *p* = 0.8780, anesthesia+ketamine *p* = 0.5268; Figure 3B), or premature responses (anesthesia+saline *p* = 0.3535, room air+saline *p* = 0.5068, anesthesia+ketamine *p* = 0.1659; Figure 3C).

### Effect of General Anesthesia on % Accuracy Over 1 Week

**FIGURE 4:**
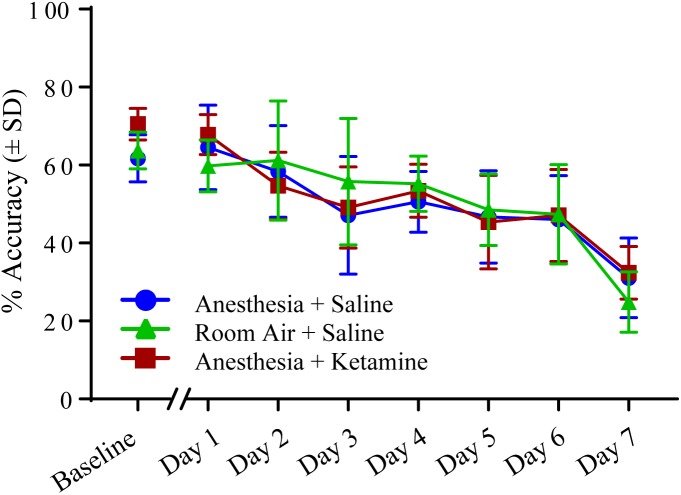
Percent accuracy measured with 5-CSRTT for 7 days following manipulation. No significant differences were detected using a two-way repeated measures ANOVA.

Figure 4 illustrates the effect of anesthesia on % accuracy across all treatment groups over the course of the experiment. (Data, shown as values (±SEM), can be seen in Supplementary Figure 1. Data analyzed in the conventional way are shown in Supplementary Figure 2). One-way ANOVA for baseline values revealed a significant difference [*F*(2, 15) = 5.129; *p* = 0.0201] between anesthesia+saline and anesthesia+ketamine (*p* = 0.0207), as shown by Tukey *post hoc* test. Two-way repeated measures ANOVA for post-manipulation comparisons showed no significant difference for treatment [*F*(2, 15) = 0.03625; *p* = 0.9645] or interaction [*F*(12, 90) = 1.126; *p* = 0.3496], but did show a difference for time [*F*(6, 90) = 34.79; *p* < 0.0001], which reflects the overall decrease in % accuracy over time for all three treatment groups. Raw data for correct and incorrect responses are shown in Table 1. Furthermore, mean correct response latency data are listed in Table 2.

**Table 1:** Attention assessment.

Attention assessment	Treatment	Baseline	Day 1	Day 2	Day 3	Day 4	Day 5	Day 6	Day 7
Correct responses	Anesthesia + Saline	46 ± 3.74	45.5 ± 4.42	41.83 ± 9.06	35 ± 8.81	37.83 ± 6113	37 ± 8.15	34.83 ± 7.73	24.17 ± 7.25
	Room air + Saline	43.5 ± 2.51	42.83 ± 3.25	40.83 ± 8.03	39 ± 9.69	37.83 ± 4.92	33.5 ± 6.66	33.17 ± 8.42	18 ± 6.39
	Anesthesia + Ketamine	49 ± 2.28	44.5 ± 3.62	39 ± 6.13	35.17 ± 5.34	36.67 ± 4.59	33.17 ± 6.43	34.5 ± 5.86	23.83 ± 4.92
Incorrect responses	Anesthesia + Saline	9.83 ± 2.48	10.5 ± 4.93	12.17 ± 6.76	19.17 ± 8.23	17.67 ± 7.42	19.83 ± 6.71	21.5 ± 5.36	28 ± 5.66
	Room air + Saline	8.18 ± 2.64	7.67 ± 3.78	10.5 ± 4.76	13.67 ± 7.97	11.83 ± 3.31	14.83 ± 4.79	13.67 ± 3.14	26.17 ± 8.03
	Anesthesia + Ketamine	7.33 ± 4.08	12.33 ± 7.79	12.33 ± 6.65	16.67 ± 5.28	18.5 ± 10.89	19 ± 8.25	20.5 ± 10.86	25 ± 7.59
Omissions	Anesthesia + Saline	4.17 ± 2.23	4 ± 3.41	6 ± 3.9	5.83 ± 4.79	4.5 ± 2.07	3.17 ± 3.31	3.67 ± 4.5	7.83 ± 7.25
	Room air + Saline	8.33 ± 2.07	9.5 ± 4.32	8.67 ± 4.84	7.33 ± 4.18	10.33 ± 4.8	11.67 ± 7.12	13.17 ± 9.28	15.83 ± 10.63
	Anesthesia + Ketamine	3.67 ± 3.5	6 ± 8.41	8 ± 5.83	7.17 ± 5.56	7.67 ± 6.62	7.33 ± 5.64	6 ± 5.73	6.67 ± 5.35
Premature responses	Anesthesia + Saline	15 ± 6.51	11.67 ± 9.83	12 ± 5.18	16.5 ± 10.84	15.17 ± 8.45	20.67 ± 13.03	16.17 ± 6.76	18.33 ± 8.09
	Room air + Saline	8.33 ± 2.25	12.33 ± 9.42	7.5 ± 4.04	8.83 ± 3.97	11.33 ± 8.64	9 ± 4.05	10.67 ± 9	11.67 ± 7.39
	Anesthesia + Ketamine	9.67 ± 5.46	5.67 ± 3.44	11.5 ± 6.59	12.5 ± 5.925	8.83 ± 5.71	15 ± 13.62	15.67 ± 16.6	13.67 ± 4.08

*Data shown as raw mean values ± SD.*

**Table 2:** Attention assessment – latencies.

Attention assessment	Treatment	Baseline	Day 1	Day 2	Day 3	Day 4	Day 5	Day 6	Day 7
Reward collection latency	Anesthesia + Saline	1.17 ± 0.311	1.24 ± 0.39	1.14 ± 0.26	1.13 ± 0.32	1.12 ± 0.23	1.12 ± 0.2	1.09 ± 0.22	1.16 ± 0.47
	Room air + Saline	1.42 ± 0.51	1.48 ± 0.52	1.46 ± 0.5	1.51 ± 0.4	1.44 ± 0.49	1.34 ± 0.52	1.41 ± 0.47	1.28 ± 0.5
	Anesthesia + Ketamine	1.19 ± 0.14	1.29 ± 0.14	2.16 ± 2.45	1.21 ± 0.14	1.2 ± 0.16	1.37 ± 0.56	1.13 ± 0.16	1.06 ± 0.15
Mean correct response latency	Anesthesia + Saline	0.83 ± 0.17	0.81 ± 0.23	0.76 ± 0.09	0.79 ± 0.19	0.83 ± 0.09	0.78 ± 0.16	0.75 ± 0.1	0.79 ± 0.18
	Room air + Saline	1.01 ± 0.07	0.96 ± 0.2	0.95 ± 0.18	0.98 ± 0.17	0.98 ± 0.27	1 ± 0.17	0.95 ± 0.31	0.95 ± 0.18
	Anesthesia + Ketamine	0.84 ± 0.09	0.95 ± 0.11	0.92 ± 0.17	0.99 ± 0.19	0.86 ± 0.16	0.8 ± 0.1	0.87 ± 0.16	1 ± 0.22

*Data shown as mean values (sec) ± SD.*

### Effect of General Anesthesia in % Omission Over 1 Week

**FIGURE 5:**
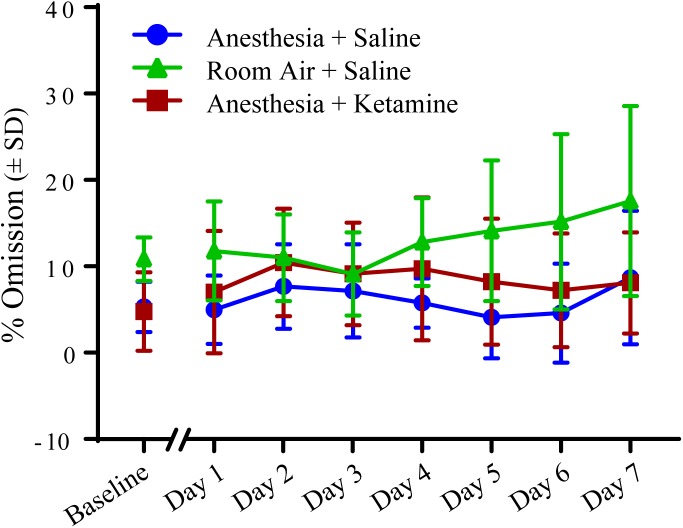
Percent omission measured with 5-CSRTT for 7 days following manipulation. No significant differences were detected using a two-way repeated measures ANOVA.

Figure 5 depicts the effect of isoflurane on % omission between all treatment groups over the course of the experiment. (Data, shown as values (±SEM), can be seen in Supplementary Figure 1. Data analyzed in the conventional way are shown in Supplementary Figure 2). One-way ANOVA for baseline value comparison showed a significant difference [*F*(2, 15) = 5.721; *p* = 0.0142] between room air+saline and anesthesia+saline (*p* = 0.0349) and room air+saline and anesthesia+ketamine (*p* = 0.0207), as revealed by Tukey *post hoc* comparison. Two-way repeated measures ANOVA for treatment groups after manipulation showed no significant difference for treatment [*F*(2, 15) = 2.889; *p* = 0.0868], time [*F*(6, 90) = 1.116; *p* = 0.3592], or interaction [*F*(12, 90) = 1.263; *p* = 0.2547]. Raw data for omissions can be found in Table 1.

### Effect of General Anesthesia on Premature Responses Over 1 Week

**FIGURE 6:**
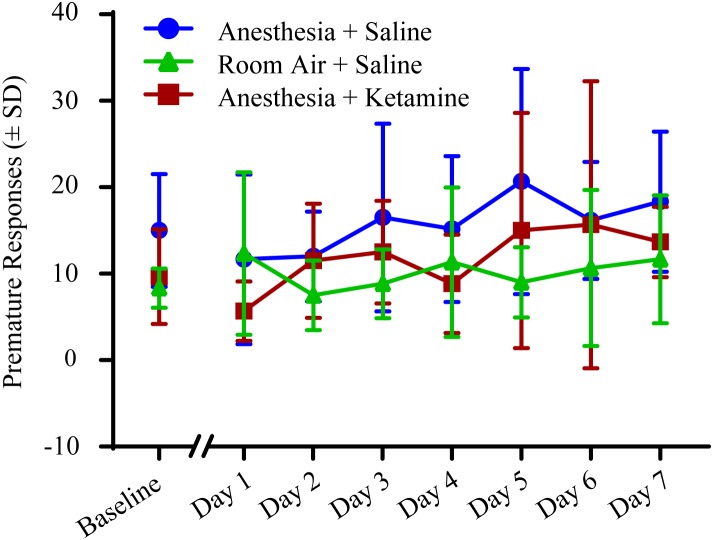
Premature responses measured with 5-CSRTT for 7 days following manipulation. No significant differences were detected using a two-way repeated measures ANOVA.

The effect of anesthesia on premature responses is shown in Figure 6. (Data, shown as values (±SEM), can be seen in Supplementary Figure 1). One-way ANOVA did not show a significant difference in baseline values between the three treatment groups [*F*(2, 15) = 2.897; *p* = 0.0864]. Two-way repeated measures ANOVA for post-treatment comparison revealed no significant difference in treatment [*F*(2, 15) = 2.55; *p* = 0.1113], time [*F*(6, 90) = 1.176; *p* = 0.3261], or interaction [*F*(12, 90) = 0.6599; *p* = 0.7849]. Raw data for premature responses are shown in Table 1. In addition, reward collection latency data are listed in Table 2.

## Discussion

The current study showed that 2 h of isoflurane anesthesia did not impair the ability to perform an attentional task for 7 subsequent days. Furthermore, the data show that adding a subanesthetic dose of ketamine to isoflurane anesthesia, which has previously been shown to accelerate recovery (Hambrecht-Wiedbusch et al., 2017), did not affect post-anesthetic cognition. Both findings suggest that isoflurane anesthesia, with or without ketamine, has no persistent effect on attention for the analyzed groups.

Surgery with anesthesia has been associated with cognitive dysfunction (Francis et al., 1990; Marcantonio et al., 1998; Lowery et al., 2008; Hudetz et al., 2009a; Carr et al., 2011; Cui et al., 2017) and, specifically, impaired attention (Ren et al., 2015; Chen et al., 2016). Disturbance of attention is a major clinical diagnostic feature in delirium in humans (Meagher et al., 2007; American Psychiatric Association, 2013; World Health Organization, 2018) and is typically identified the day after surgery (Bilotta et al., 2016; Cui et al., 2017). In this study, comparing cognitive behaviors between the day before anesthesia treatment (baseline) with the day after treatment (day 1) did not show any significant differences for % accuracy, % omission, or premature responses between exposure to isoflurane anesthesia vs. room air.

When evaluating % accuracy, which can be seen as a measure of memory for task recall, animals treated with anesthesia did not differ on any of the days after manipulation from animals treated with room air, confirming that the animals had no impaired cognition and were able to recall the previously learned task. This is similar to a study in humans by Chen et al. (2016), in which the attention network task was used to examine the efficiency of the alerting, orienting, and executive control attention networks in middle aged women undergoing gynecologic surgery. Using the 5-CSRTT, another aspect of attention was evaluated, % omission, which tests the inability to perform the task and can shed light on sensory, motor, or motivational factors. Overall, there was no significant difference in % omission for all three treatment groups over the 7 days time period. The slight, but non-significant, increase in room air treated animals on the last testing days is likely due to the fact that one animal was showing relatively more omissions at the end of the week, probably because the animal was not seeing the light cue while still sniffing in and around the food tray area (see Supplementary Figure 3). Another measure of cognitive impairment is premature response, which is a surrogate for the impulsivity of an animal and the inability to focus attention and wait for the correct cue. The results of this study are similar to the work of Carmen et al. (2016), in which anesthesia did not alter the impulsivity to perform a task. Our results show that exposure to isoflurane anesthesia does not appear to be a confound for experiments involved in attention.

The unique anesthetic drug ketamine is regularly used in clinical settings and is advantageous because it maintains respiratory drive and airway patency (Eikermann et al., 2012). Hudetz et al. (2009a,c) found that low-dose ketamine improved cognition 1 week after cardiac surgery and reduced post-operative delirium. However, in the current study, a subanesthetic dose of ketamine given during exposure to isoflurane did not change performance (% accuracy, % omission, and premature responses) of attentional tasks. This is consistent with results of the multicenter PODCAST trial by Avidan et al. (2017), in which intraoperative, subanesthetic ketamine did not change the incidence of delirium in humans. It is also consistent with a study by Bilotta et al. (2016), who showed that ketamine given during general anesthesia in humans did not cause post-operative cognitive changes.

### Strengths and Limitations

One strength of this study is that it evaluated general anesthesia without the influence of surgery, pain (Heyer et al., 2000; Cheng et al., 2008; Zywiel et al., 2014), inflammation (Wan et al., 2007; Caza et al., 2008; Li et al., 2012; van Harten et al., 2012; Hovens et al., 2014), and drug interactions (Burns et al., 1990; Francis et al., 1990; Millar, 1998; Moore and O’Keeffe, 1999). Another strength is that the study specifically evaluated attention, which is arguably more relevant to delirium than spatial memory. Evaluating attention daily over the course of a week was also a methodologic strength because it is similar to the time course of post-operative delirium, since delirium in humans can persist beyond post-operative day 1. Therefore, studies have evaluated cognition several days to a week after surgery (Francis et al., 1990; Marcantonio et al., 1998; Lowery et al., 2008; Hudetz et al., 2009b,c; Carr et al., 2011; Cui et al., 2017).

Although it could be argued that having the animals perform the same test for 7 consecutive days would improve performance through a learning effect, the stimulus duration was reduced daily and the program still randomly chose which grid was illuminated, which forced the animal to engage attention and precluded pattern detection. The overall decrease of the % accuracy values over time provides evidence that the reduced stimulus duration made the task harder. This is confirmed by Higgins and Silenieks (2017), who showed that changing the stimulus duration is correlated with the % correct responses, meaning shorter stimulus duration results in reduced % accuracy. Methodologic weaknesses include the restriction to healthy non-elderly animals, the single halogenated ether used, and the relatively short (although still clinically relevant) exposure to isoflurane. Furthermore, it is possible that this particular performance task was not sensitive enough to detect subtler attentional deficits. Finally, there are no well-defined rodent models of delirium and thus the translational relevance of these findings to humans must be established through further research.

## Summary

Collectively, these data suggest that – in healthy animals that are not undergoing surgery – general anesthesia alone does not have a persistent effect on attention. Impaired cognition after surgery might mainly depend on other factors such as surgery, inflammation, other drugs, co-morbid conditions, and age.

## Ethics Statement

This study was carried out in accordance with the recommendations of the Guide for the Care and Use of Laboratory Animals (National Academies Press, 8th Edition, Washington, DC, 2011) and the Institutional Animal Care and Use Committee (IACUC) supported by the Animal Care and Use Office. The protocol was approved by the IACUC.

## Author Contributions

VH-W designed the study, conducted experiments, analyzed data, and wrote the manuscript. KL and RA conducted experiments and analyzed data. MA consulted on experimental design and revised the manuscript. AN and MP conducted experiments, analyzed data, and participated in drafting the manuscript. GM designed the study, interpreted data, and wrote the manuscript. All authors listed have made a substantial, direct and intellectual contribution to the work, and approved it for publication.

## Conflict of Interest Statement

The authors declare that the research was conducted in the absence of any commercial or financial relationships that could be construed as a potential conflict of interest.
