# Supplementary material for: General Anesthesia Does Not Have Persistent Effects on Attention in Rodents
Source: Front Behav Neurosci. 2019 Apr 17;13:76. doi: 10.3389/fnbeh.2019.00076 (PMC6478802; doi:10.3389/fnbeh.2019.00076)
Supplement: Supplementary file 1 [file Data_Sheet_1.docx]

.
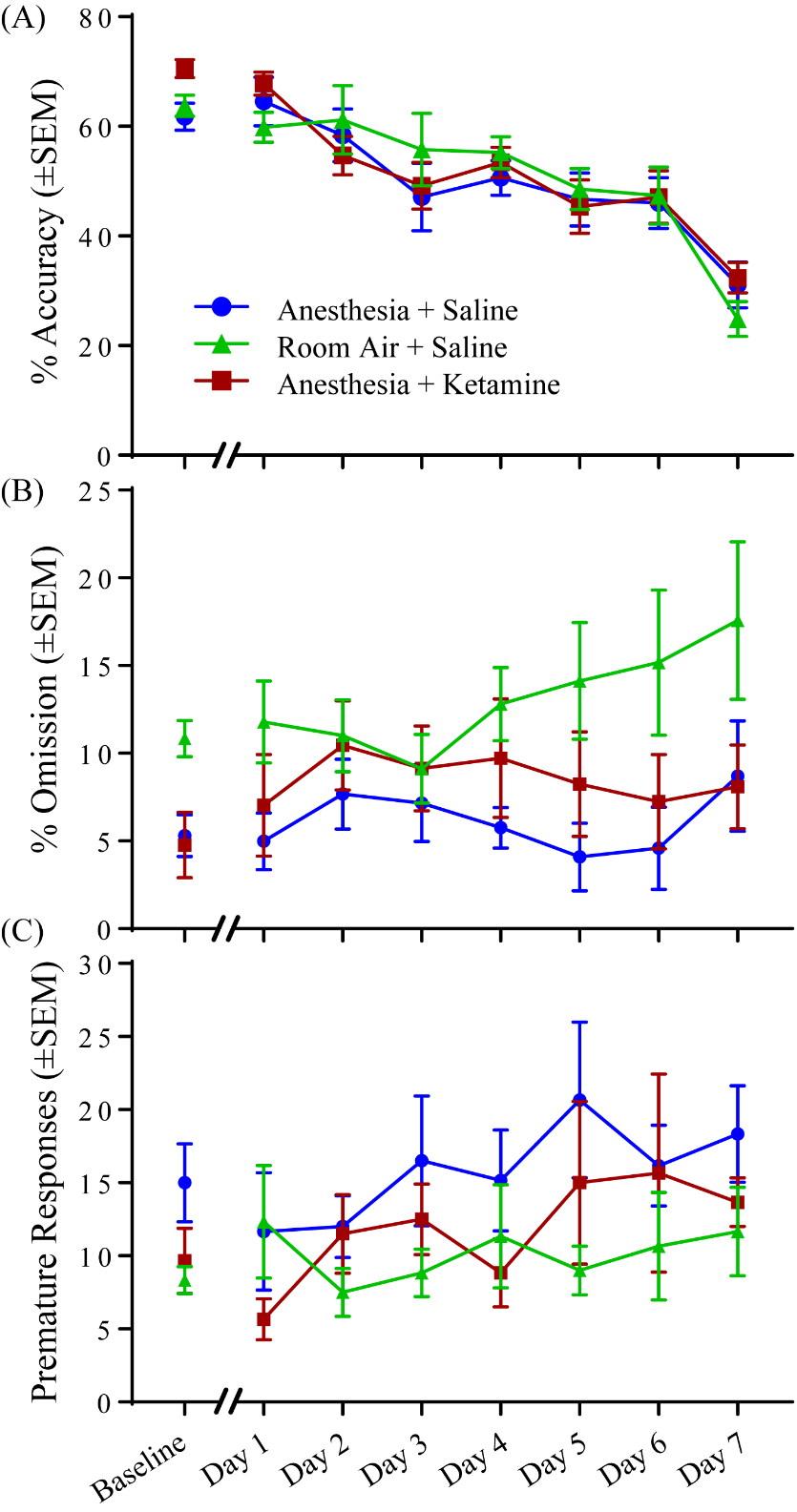


**Supplementary Figure 1:** Percent accuracy, % omission, and premature responses shown as mean ± SEM measured with the 5-CSRTT for 7 days following manipulations.
