# Supplementary material for: General Anesthesia Does Not Have Persistent Effects on Attention in Rodents
Source: Front Behav Neurosci. 2019 Apr 17;13:76. doi: 10.3389/fnbeh.2019.00076 (PMC6478802; doi:10.3389/fnbeh.2019.00076)
Supplement: Supplementary file 2 [file Data_Sheet_2.docx]

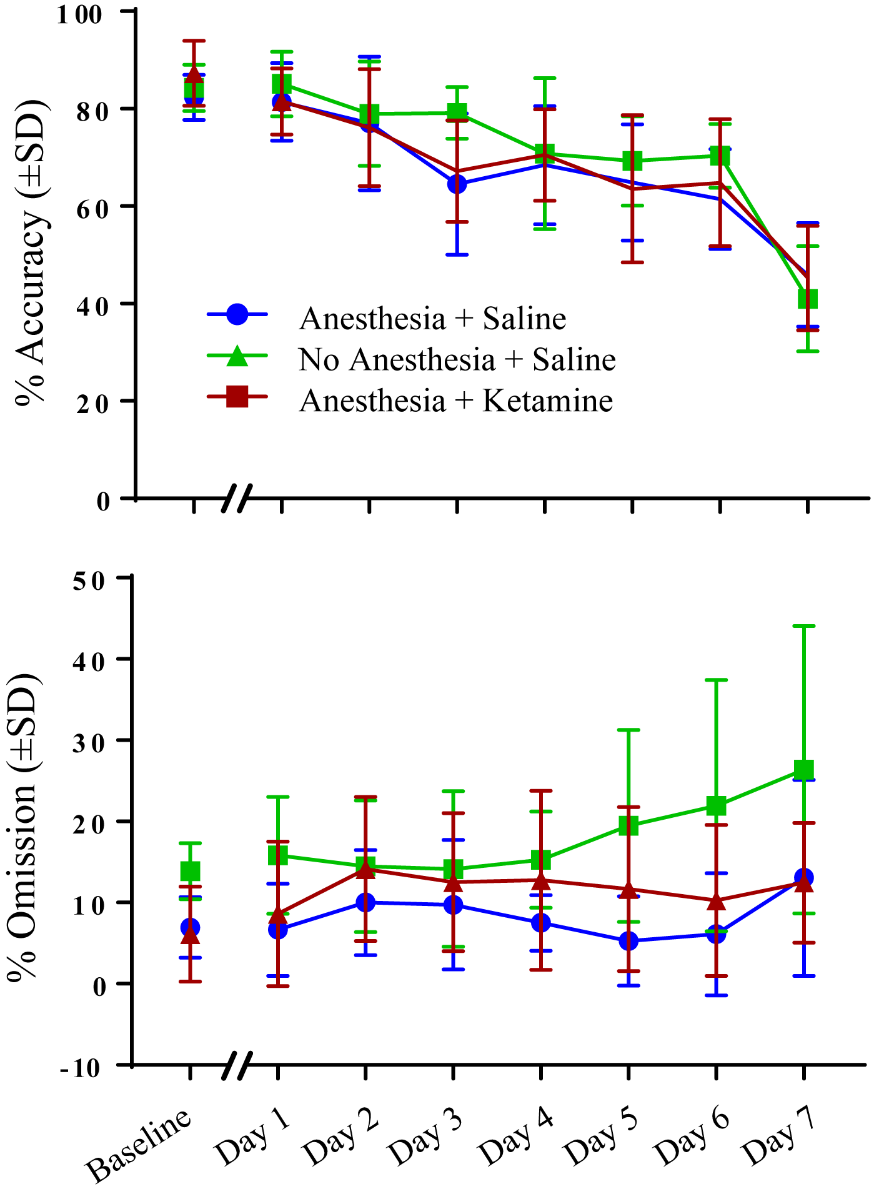


**Supplementary Figure 2:** Percent accuracy and % omission (± SD) measured with the 5-CSRTT for 7 days following manipulations using conventional measurements, which means no inclusion of omissions and premature responses into total number of trials. For % accuracy, one-way ANOVA for baseline comparisons showed no significant difference between the three treatment groups [F(2, 15) = 1.26; p = 0.3121]. A two-way repeated measures ANOVA showed no significant differences in post-manipulation for treatment [F(2, 15) = 0.5153; p = 0.6075] and interaction [F(12, 90) = 1.009; p = 0.4478], but a significant difference in time [F(6, 90) = 41.32; p < 0.0001], which reflects the overall decrease in % accuracy over time for all three treatment groups. For % omission, one-way ANOVA for baseline comparisons showed a significant difference [F(2, 15) = 5.48, p = 0.0163] between room air+saline and anesthesia+saline (p = 0.0418) and between room air+saline and anesthesia+ketamine (p = 0.0223). Two-way repeated measures ANOVA for post-manipulations did not show a significant difference for treatment [F(2, 15)=2.825; p=0.0910], time [F(6, 90) = 1.908; p = 0.0880], and interaction [F(12, 90) = 1.227; p = 0.226].
