# Supplementary material for: General Anesthesia Does Not Have Persistent Effects on Attention in Rodents
Source: Front Behav Neurosci. 2019 Apr 17;13:76. doi: 10.3389/fnbeh.2019.00076 (PMC6478802; doi:10.3389/fnbeh.2019.00076)
Supplement: Supplementary file 3 [file Data_Sheet_3.docx]

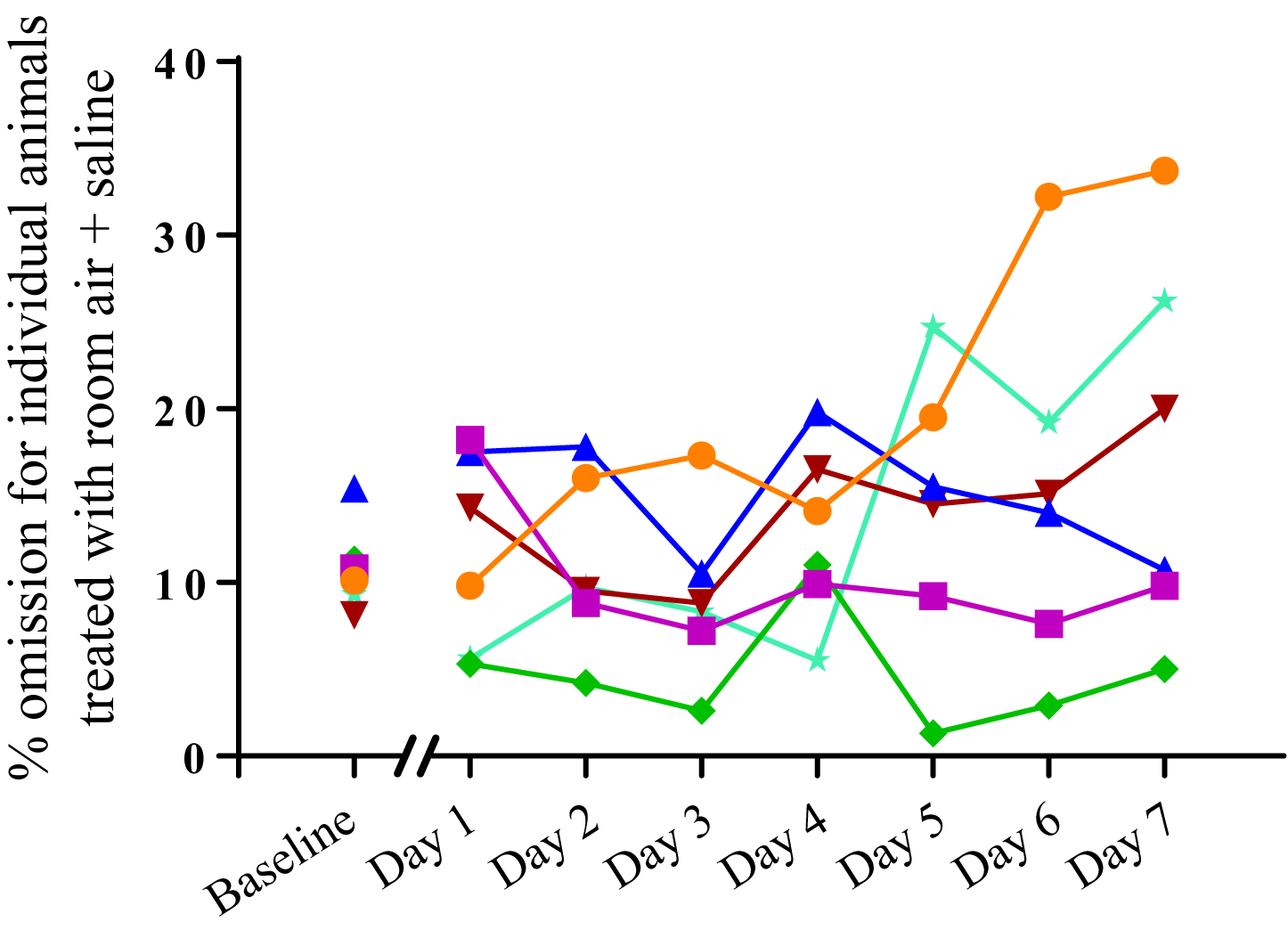


**Supplementary Figure 3:** Percent omission for six individual animals treated with room air+saline measured with the 5-CSRTT for 7 days following manipulation.
